# Identification of a strawberry *NPR*-like gene involved in negative regulation of the salicylic acid-mediated defense pathway

**DOI:** 10.1371/journal.pone.0205790

**Published:** 2018-10-12

**Authors:** Lin-Jie Shu, Jui-Yu Liao, Nai-Chun Lin, Chia-Lin Chung

**Affiliations:** 1 Department of Plant Pathology and Microbiology, National Taiwan University, Taipei, Taiwan; 2 Department of Agricultural Chemistry, National Taiwan University, Taipei, Taiwan; Fujian Agriculture and Forestry University, CHINA

## Abstract

Hormonal modulation plays a central role in triggering various resistant responses to biotic and abiotic stresses in plants. In cultivated strawberry (*Fragaria* x *ananassa*), the salicylic acid (SA)-dependent defense pathway has been associated with resistance to *Colletotrichum* spp. and the other pathogens. To better understand the SA-mediated defense mechanisms in strawberry, we analyzed two strawberry cultivars treated with SA for their resistance to anthracnose and gene expression profiles at 6, 12, 24, and 48 hr post-treatment. Strawberry genes related to SA biosynthesis, perception, and signaling were identified from SA-responsive transcriptomes of the two cultivars, and the induction of 17 candidate genes upon SA treatment was confirmed by qRT-PCR. Given the pivotal role of the non-expressor of pathogenesis-related (NPR) family in controlling the SA-mediated defense signaling pathway, we then analyzed *NPR* orthologous genes in strawberry. From the expression profile, *FaNPRL-1* [ortholog of *FvNPRL-1* (*gene20070* in *F*. *vesca*)] was identified as an *NPR*-like gene significantly induced after SA treatment in both cultivars. With a conserved BTB/POZ domain, ankyrin repeat domain, and nuclear localization signal, FvNPRL-1 was found phylogenetically closer to NPR3/NPR4 than NPR1 in Arabidopsis. Ectopic expression of *FvNPRL-1* in the *Arabidopsis thaliana* wild type suppressed the SA-mediated *PR1* expression and the resistance to *Pseudomonas syringae* pv. *tomato* DC3000. Transient expression of FvNPRL-1 fused with green fluorescent protein in Arabidopsis protoplasts showed that SA affected nuclear translocation of FvNPRL-1. FvNPRL-1 likely functions similar to Arabidopsis NPR3/NPR4 as a negative regulator of the SA-mediated defense.

## Introduction

Cultivated strawberry (*Fragaria* x *ananassa*) is one of the most economically important Rosaceae fruit crops. Genetically, *F*. x *ananassa* is a complex allo-octoploid plant derived from two diploid species, *F*. *virginiana* and *F*. *chiloensis*. The genome of the diploid woodland strawberry *F*. *vesca* (~240 Mb) was released in 2011, providing a good resource for studying *Fragaria* spp. [1]. Cultivated strawberry is subject to the infestation of various phytopathogenic organisms. Major fungal diseases of strawberry include anthracnose, gray mold, powdery mildew, *Phytophthora* fruit rot, bacterial wilt, *Fusarium* wilt, and foliar nematode [2]. Because pest damage and lack of resistant cultivars are major challenges for strawberry cultivation, exploring the defense mechanism in cultivated strawberry may provide insights into the development of new disease control strategies.

Phytohormones are involved in modulating various plant responses to biotic and abiotic stresses [3]. In many model plants, salicylic acid (SA) is a critical defense signaling molecule in plant immunity against biotrophic and hemibiotrophic pathogens [3]. NahG transgenic Arabidopsis, which expressed bacterial salicylate hydroxylase and accumulated less SA, was susceptible to infection with *Peronospora parasitica* [4], *Colletotrichum destrctivum* [5], and *Pseudomoans syringae* [6]. SA was required for inducing systemic acquired resistance (SAR), which involves the upregulation of a suite of pathogenesis-related (*PR*) genes [7, 8]. In cultivated strawberry, exogenous application of SA led to increased resistance to *C*. *acutatum* and *C*. *gloeosporioides* [9, 10]; elevated levels of SA and *PR* gene transcripts were detected after infection with *C*. *acutatum*, *C*. *fragariae*, and *C*. *gloeosporioides* [10–12]. A recent microarray study of strawberry responses to *C*. *acutatum* also revealed the induction of transcripts corresponding to SA and jasmonic acid (JA) signaling pathways [12]. These findings indicate the existence of the SA-dependent defense signaling pathway and its importance in disease resistance in strawberry.

In Arabidopsis, transcription factors and regulators such as non-expressor of pathogenesis-related (NPR) [13], TGACGTCA *cis*-element-binding protein (TGA) [14, 15], WRKY DNA-binding protein (WRKY; containing highly conserved amino acid sequences WRKYGQK, namely WRKY domain) [16, 17], and glutaredoxin (GRX) [18] play important roles in modulating the balance and crosstalk among distinct defense pathways. NPR1 is a positive regulator controlling the SA-mediated defense responses and induced systemic resistance [7, 19–21]. NPR1 possesses an N-terminal BTB/POZ domain, an ankyrin repeat, and a C-terminal nuclear localization signal (NLS) [22–24]. In naïve tissues, NPR1 is present predominantly in an oligomeric form in the cytosol. Upon pathogen infection or SA treatment, increased SA content induces thioredoxin-mediated reduction of cysteine 156, thereby leading to the release of monomers from the oliogomeric NPR1 complex. The NPR1 monomer can translocate into the nucleus [22], where it interacts with co-activators such as TGA, NIM1-INTERACTING (NIMIN) proteins, and WRKY transcription factors, thereby increasing the expression of defense-related genes [20, 25]. However, the NPR1 paralogs NPR3 and NPR4 function as negative regulators of SA-mediated immunity [26]. Independent studies showed that SA binding to NPR3/NPR4 regulated the transcription of SA-responsive defense genes by interacting with NPR1 for proteasome degradation [27–29] or by independently interacting with TGA2/TGA5/TGA6 transcription factors [30]. Recently, NPR3 and NPR4 were also found as regulators of JA synthesis and signaling pathways [31]. GRXs, belonging to another group of early SA-inducible genes in Arabidopsis, encode glutaredoxins that mediate redox regulation of proteins involved in SA-mediated defense. Via interactions with TGA factors, GRXs also participate in SA–JA cross-talk [18, 32, 33].

To better understand the SA-mediated defense mechanisms in strawberry, we analyzed two strawberry cultivars for their resistance to anthracnose and gene expression profiles after SA treatment. Illumina sequencing and time-course qRT-PCR analyses revealed a set of 17 genes including known and potential defense-related genes with differential expression upon SA treatment, which suggests their involvement in SA-mediated defense responses in strawberry. Among these genes, *gene20070* encodes a protein belonging to one of the NPR family members in strawberry (NPR-like genes in *F*. *vesca*; *FvNPRLs*), which suggests its potential role as a master regulator of SA-mediated defense responses. The functions of *NPR1* orthologues in many crops [20] and potential roles of *NPR3* orthologues in cacao and *NPR3*/*NPR4* in citrus have been uncovered [34, 35], but little is known about *FvNPRLs*. In the diploid strawberry genome sequence database [1], five *FvNPRL* genes were found, but none have been further characterized. We only know that overexpression of *AtNPR1* in cultivated strawberry increased its resistance to anthracnose, powdery mildew, and angular leaf spot [36]. In this study, phylogenetic analysis and functional characterization revealed the role of *FvNPRL-1* (*gene20070*). The results suggest that similar to *NPR3*/*NPR4*, *FvNPRL-1* may translocate into the nucleus and function as a negative regulator of the defense responses against biotic stresses.

## Materials and methods

### Plant materials and growth conditions

Two cultivated strawberry (*F*. *x ananassa*) cultivars, Taoyuan no. 3 (TY3) and Superjumbo (SJ), and the woodland strawberry (*F*. *vesca* var. *vesca*) were used in this study. The strawberry mother plants were cultured in soil mix (peat: perlite: vermiculite, 1: 1: 1, v/v/v) under 16-hr photoperiod at 24°C. The daughter plants were reproduced from the mother plants by stolon propagation.

For cultivation of *Arabidopsis thaliana* ecotype Col-0 wild type and transgenic lines, seeds were sterilized with 75% ethanol and 2% bleach, then grown on Murashige-Skoog (MS) medium (4.3 g/L MS salt, pH 5.7, 1% sucrose, and 0.4% phytagel) for 12 days. Seedlings were transferred to soil mix (peat: perlite: vermiculite, 9: 1: 1, v/v/v) in 3-inch–diameter plastic pots and grown under 16-hr photoperiod at 22°C.

### SA treatment

Strawberry seedlings at the 4–5 leaf stage were sprayed with 5 mM SA or sterile ddH_2_O by airbrush (4 ml per plant, 10–15 psi) and kept in an incubator for 2 days under >90% relative humidity (RH) at 24°C and 16-hr photoperiod. For Arabidopsis, 12-day-old seedlings were transferred to MS medium containing 200 μM SA and incubated for 24 hr under a 16-hr photoperiod at 22°C. Seedlings transferred to MS medium without SA were used as control plants.

### Evaluation of anthracnose resistance

Resistance to anthracnose was evaluated with a *C*. *gloeosporioides* isolate GL001 collected from an infected strawberry leaf from Miao-Li, Taiwan, in 2011. The inoculum was cultured on 1/4 potato dextrose agar (PDA) under a 12-hr photoperiod at room temperature for 7–10 days. Inoculation was conducted 72 hr after SA or ddH_2_O treatment by spraying the entire strawberry plants with 2–3 ml conidial suspension per plant (1 x 10^6^ spores/ml in 0.02% Tween 20) with use of an airbrush at 10–15 psi. Sterilized ddH_2_O containing 0.02% Tween 20 was used as the control. The inoculated plants were kept in plastic boxes for 24 hr at >90% RH and 25°C, then maintained in a growth chamber at 25°C, 60% RH under an 8-hr photoperiod. Disease severity was evaluated at 3, 4, and 5 days post-inoculation (dpi). Individual plants were scored by using a self-defined disease severity index (DSI) with a 1–10 rating scale (S1 Table). The experiment was performed in three independent trials, each containing 3–4 plants per cultivar per treatment. Phenotypic differences were analyzed by Tukey's Honestly Significant Difference (HSD) test at *p* < 0.05 with SAS/STAT v9.3 (SAS Institute Inc., Cary, NC, USA).

### RNA extraction and quantitative real-time RT-PCR

Strawberry leaf samples and Arabidopsis leaf/seedling samples were collected, snap-frozen in liquid nitrogen, and ground into fine powder in liquid nitrogen. Total RNA was extracted from strawberry and Arabidopsis by using the cetyltrimethylammonium bromide (CTAB)-based extraction method [37] and TRIzol Reagent (Life Technologies), respectively. To eliminate DNA contamination, strawberry and Arabidopsis RNA samples were treated with a TURBO DNase Kit (Ambion) following the manufacturer’s instructions. The concentration of each RNA sample was measured by using the Nanodrop Spectrophotometer ND-100, then cDNA was synthesized by using the MMLV Reverse Transcriptase cDNA Synthesis Kit (Epicentre) following the manufacturer’s instructions.

Each strawberry or Arabidopsis experiment involved 2–3 independent trials, with 3–4 strawberry plants or 3 Arabidopsis plants per treatment per trial. Relative gene expression was measured in three technical replicates with the StepOnePlus Real-Time PCR System (Applied Biosystems). Each qRT-PCR reaction contained 10 μl Fast SYBR Green Master Mix, 1 μl of 10 μM forward primer, 1 μl of 10 μM reverse primer, 0.5 μl cDNA, and 7.5 μl ddH_2_O. The primers listed in S2 Table and S3 Table for qRT-PCR were designed to amplify 100- to 150-bp fragments of targeted genes. qRT-PCR was performed under thermal cycling parameters of 95°C for 20 sec followed by 40 cycles of 3 sec at 95 ^o^C and 30 sec at 60°C. The strawberry housekeeping gene *FaActin* and Arabidopsis *AtActin2* were internal controls. The cycle threshold (C_T_) values for each gene in different samples were normalized to C_T_ values of the internal control gene in a given sample. The relative gene expression in different samples was calculated by the ΔΔC_T_ method [38].

### Transcriptome analysis

Equal amounts of total RNA from 6, 12, 24, and 48 hr post-SA or ddH_2_O treatment were pooled for strand-specific RNA sequencing (2 x 101 bp paired-end reads) on the Illumina HiSeq 2000 at Yourgene Bioscience (Taipei, Taiwan). At each time point, RNA was isolated from the first fully expanded leaves collected from 3–4 strawberry seedlings. RNA-seq data have been deposited in the NCBI Short Read Archive database under the accession numbers SRX4076953 (SJ—SA treatment), SRX4076954 (SJ—Control), SRX4076955 (TY3—SA treatment), and SRX4076956 (TY3—Control). The trimmed and filtered reads (error probability < 0.01, Q20; read length > 35 bp) from the same cultivar were first *de novo* assembled by the Trinity method (version r20121005) [39], then mapped to the diploid woodland strawberry database *Fragaria vesca* Whole Genome v1.0 (build 8) [1, 40, 41] by using Bowtie 2 [42]. Differential expression analysis was conducted with the R package DESeq [43]. The unigene sequences were annotated by a BLAST search of the *Fragaria vesca* Whole Genome v1.0 (build 8) database with *E*-value cutoff of 10^−5^. Confidence results of differential expression analysis were determined on the basis of FPKM values and contigs with at least 30 independent read counts from the same cultivar (from previous studies of *Fragaria* RNA-seq [37]). Less stringent criteria (fold change ≥ 1.2 and ≤ 0.8) were adopted for gene identification to gain an overview of the entire defense pathways.

### 3’ rapid amplification of cDNA ends (3’ RACE) of strawberry *FvNPRL-3*

The RNA of diploid strawberry was isolated and treated with DNase. Reverse transcription was conducted as described above, but the oligo-(dT)_20_ primer was substituted by the 3’RACE adaptor [oligo-(dT)_20_ anchored with a specific adaptor] (S3 Table). The cDNA template was used in nested-PCR for 3’ exon and 3’ UTR amplification. The forward primer targeting the 2^nd^ exon of *FvNPRL-3* and the 3’ RACE outer primer were used for the first PCR, then the forward primer targeting the 4^th^ exon of *FvNPRL-3* and the 3’ RACE inner primer were used for the second PCR. The ~500-bp PCR product was ligated into the T&A Cloning Vector (Yeastern Biotech, Taiwan) and sequenced by using M13-F and M13-R primers at the Center for Biotechnology, National Taiwan University.

### Phylogenetic analysis of *NPR*-like genes

Five *NPR-Like* (*NPRL*) genes have been annotated in the diploid strawberry genome: *gene20070*, *gene28768*, *gene28770*, *gene12668*, and *gene21905*, designated *FvNPRL*-*1*, *FvNPRL-2*, *FvNPRL-3*, *FvNPRL-4* and *FvNPRL-5*, respectively, in this study. To investigate the relation among *NPR-like* genes in strawberry and other plant species, we constructed a phylogenetic tree by using the amino acid sequences of NPR-like genes from *A*. *thaliana*, *Citrus sinensis*, *Glycine max*, *Musa acuminate*, *Oryzae sativa*, *Phalaenopsis aphrodite* subsp. *Formosana*, *Populus trichocarpa*, *Sorghum bicolor*, *Theobroma cacao*, *Vitis vinifera*, and *Zea mays*. MEGA v7.0.26 [44] was used to align sequences and the phylogenetic tree was constructed by the neighbor-joining method with 1000 bootstrap replicates.

### Construction of the binary vector carrying *FvNPRL-1* for Arabidopsis transformation

A DNA fragment of *FvNPRL-1* was amplified from *F*. *vesca* var. *vesca* with the primers *FvNPRL-1*-F and *FvNPRL-1*-R carrying *NcoI* and *PmacI* sites (S3 Table) by Phusion High-Fidelity DNA Polymerase (Thermo Scientific). The PCR product was first ligated into the T&A Cloning Vector (Yeastern Biotech, Taiwan), resulting in the plasmid pyT&A-*FvNPRL-1*. *FvNPRL-1* was released from pyT&A-*FvNPRL-1* by *NcoI*-*PmacI* partial digestion, then ligated into *Nco*I/*Pmac*I-treated pCAMBIA1301. The resulting pCAMBIA1301-*FvNPRL-1* was transformed into *Agrobacterium tumefaciens* strain GV3101. The floral dip method [45] was used to transform *Arabidopsis thaliana* Col-0 wild type, and the transformed seeds were selected on MS medium containing 50 μg/μl hygromycin B. Each transformed line was re-selected by hygromycin B for two generations to obtain homozygous seedlings. The T_2_ lines with all tested T_3_ progeny (at least 90 seeds) surviving on hygromycin B-containing plates were considered homozygous.

### *Pseudomonas* inoculation on Arabidopsis

*Pseudomonas syringae* pv. *tomato* (*Pst*) strain DC3000 was grown in King’s B medium (KBM) containing 100 μg/ml rifampicin at 28°C. Cells of *Pst* DC3000 from overnight cultures were collected, washed with sterilized 10 mM MgCl_2_ and adjusted to O.D.600 = 0.05 in 10 mM MgCl_2_ supplemented with 0.02% Silwet L-77. Arabidopsis plants at 25 days old were spray-inoculated with the bacterial suspension until water ran off and homogeneously distributed on the leaf surface. At days 0 and 3 after inoculation, the first fully expanded leaf from each plant was weighed, then homogenized in 1 ml cold 10 mM MgCl_2_. Serial dilutions were made and 10 μL of each dilution was spotted on the KBM plates containing 100 μg/ml rifampicin. After growing at 28°C for 30 hr, colony-forming units (CFU) per milligram fresh leaf tissue were calculated [46]. Each transgenic line contained 6 plants, and the inoculation assay was repeated three times.

### Subcellular localization of GFP-tagged FvNPRL-1 in Arabidopsis protoplasts

A DNA fragment of *FvNPRL-1* was amplified from *F*. *vesca* var. *vesca* with the primers *FvNPRL-1*-F and *FvNPRL-1*-R carrying attB1 and attB2 sites (S3 Table) by Phusion High-Fidelity DNA Polymerase (Thermo Fisher Scientific). The fragment was cloned into the Gateway compatible vector p2GWF7, resulting in p2GWF7-*FvNPRL-1*, which expresses FvNPRL-1 fused with GFP at its C-terminus.

The isolation and transfection of Arabidopsis protoplasts were performed following the polyethylene glycol method [47, 48]. After transfection with p2GWF7-*FvNPRL-1*, protoplasts were treated with 0, 1, 10, and 40 μM SA and harvested after incubation for 20 hr. The nucleus localization marker VirD2-NLS-mCherry was used [49]. Transfected protoplasts were examined by confocal microscopy (Zeiss LSM 510 META NLO DuoScan). GFP and mCherry in the protoplasts were excited by 488 and 543 nm laser beans and the detection spectrum ranged from 500 to 587 and 600 to 630 nm, respectively. This transient expression experiment was repeated three times.

## Results

### SA-induced resistance to anthracnose in cultivated strawberry

**Fig 1:**
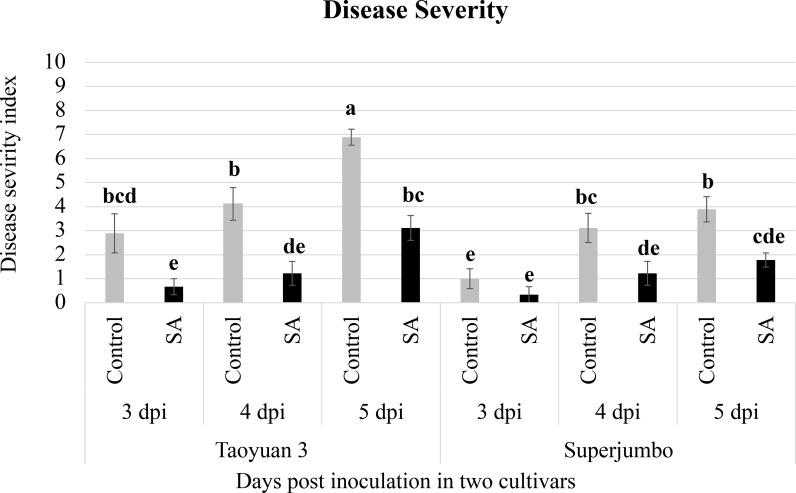
Anthracnose resistance in salicylic acid (SA)- and ddH_2_O-treated strawberry plants. To confirm that exogenous SA application can induce strawberry defense responses, plants of the cultivars Taoyuan 3 and Superjumbo were sprayed with SA or ddH_2_O and inoculated with *Colletotrichum gloeosporioides* at 72 hr after treatment. Disease severity was evaluated at 3, 4, and 5 dpi with a 1–10 scale based on S1 Table. Data are mean ± SEM (*n* = 3 independent trials and 3–4 plants per treatment per trial). Different letters indicate significant difference based on Tukey's Honestly Significant Difference (HSD) test at *p* < 0.05.

To ensure that SA-mediated defense mechanisms exist in the two selected cultivars TY3 and SJ, strawberry plants treated with 5 mM SA or ddH_2_O (control) were inoculated with *C*. *gloeosporioides* and evaluated for disease severity. At 2 dpi, disease symptoms could be observed on the leaves of ddH_2_O-treated but not SA-treated strawberry plants. Both SJ and TY3 plants treated with SA showed delayed development of anthracnose symptoms as compared with mock-inoculated plants, and SJ plants exhibited higher resistance to anthracnose than TY3 plants (Fig 1).

**Fig 2:**
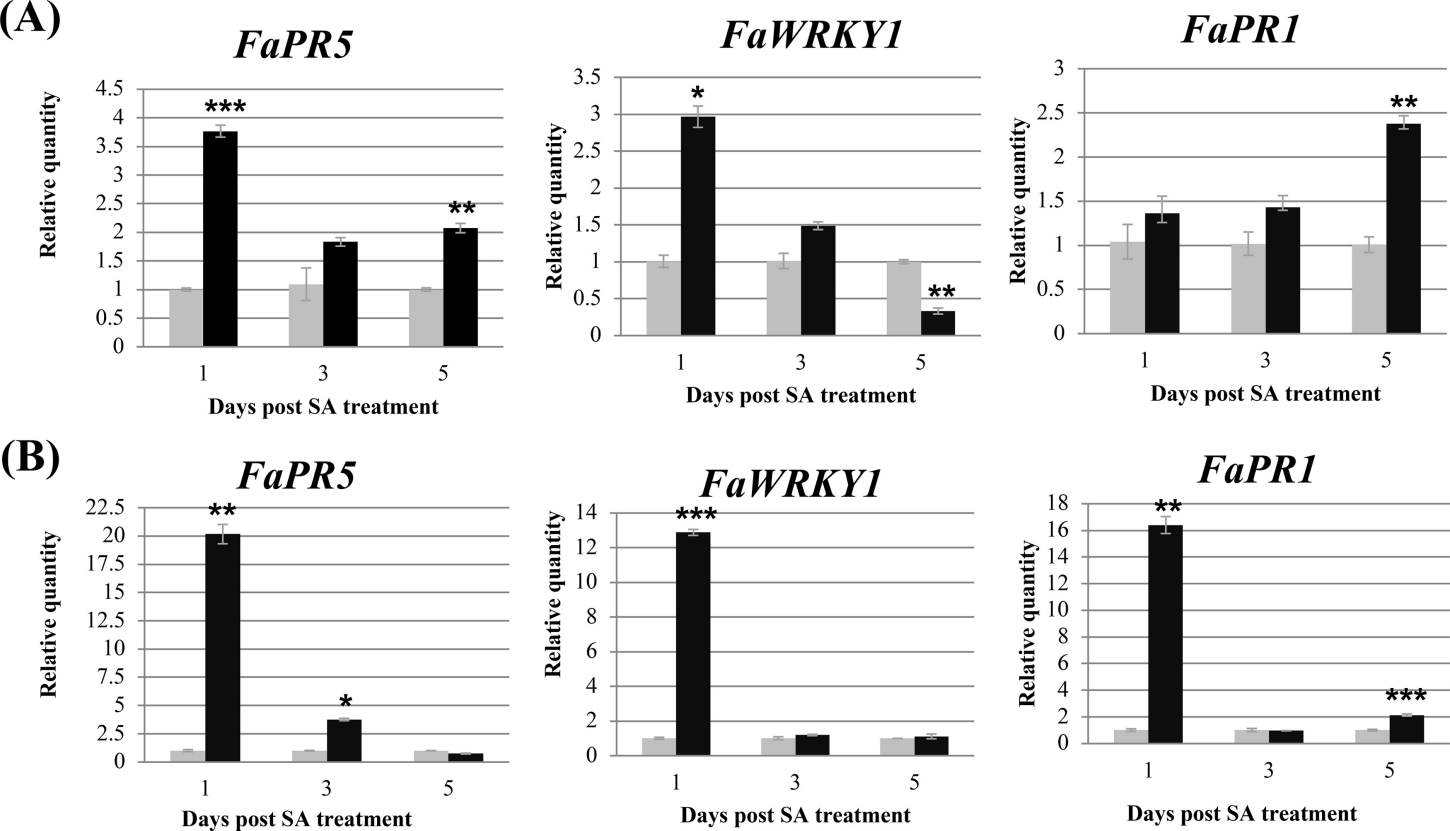
Expression profiling of three strawberry defense marker genes after salicylic acid (SA) or ddH_2_O treatment by real-time quantitative RT-PCR. The expression of *FaPR5* (*FaOLP2*), *FaWRKY1*, and *FaPR1* on 1, 3, and 5 days after SA (dark bars) or ddH_2_O (light bars) treatment was investigated in the cultivars Taoyuan no. 3 (A) and Superjumbo (B). *FaActin* was an internal control. Data are mean ± SEM (*n* = 3 independent trials and 3–4 plants per treatment per trial). Differences between the SA and ddH_2_O treatment at each time point were analyzed by two-tailed Student’s *t* test. Significance level: * *p* < 0.05, ** *p* < 0.01, *** *p* < 0.001.

Three previously characterized strawberry genes, *FaPR5* (*FaOLP2*), *FaWRKY1*, and *FaPR1* [11, 50, 51], were used as markers for detecting the activation of the SA-mediated defense pathway. In response to SA, the expression of the three genes was enhanced within 5 days [11, 50, 51]. Time-course gene expression was analyzed for the RNA samples extracted from TY3 or SJ plants on days 1, 3, and 5 after SA or ddH_2_O treatment. All three genes were triggered in both cultivars under 5 mM SA treatment, although the induction levels and expression patterns differed between the two cultivars (Fig 2). Of note, the induction of the three marker genes was faster and higher in the more resistant cultivar SJ than in TY3. The expression patterns also indicated that the SA-mediated defense pathway could be activated within 24 hr after SA treatment.

### Identification of defense-related genes differentially expressed upon SA treatment

**Fig 3:**
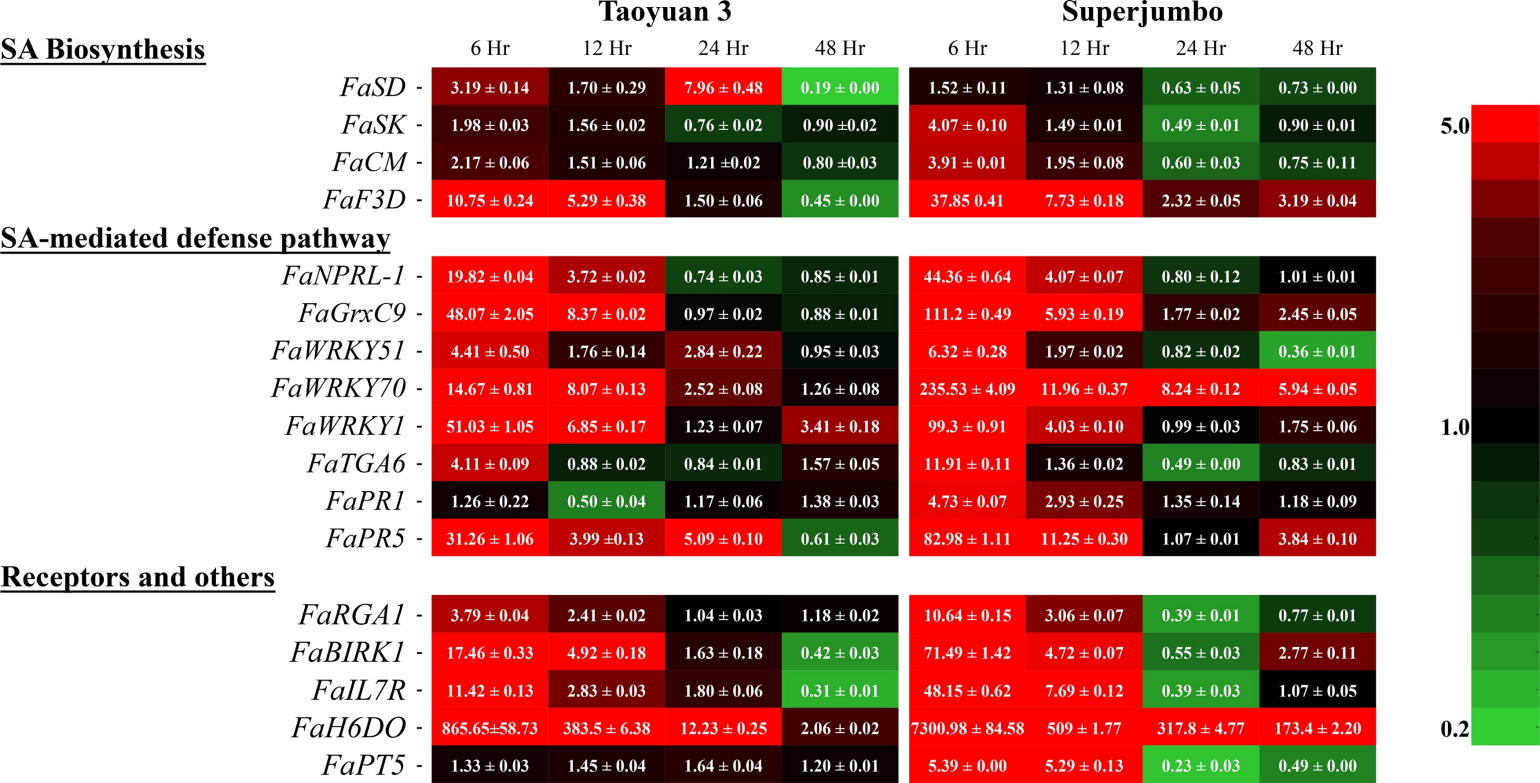
Expression profiling of strawberry genes putatively involved in the salicylic acid (SA)-mediated defense network. qRT-PCR was used to investigate the differential expression of 17 genes in strawberry cultivars Taoyuan 3 and Superjumbo at 6, 12, 24 and 48 hr after SA treatment. *FaActin* was an internal control. *FaSD*: *shikimate dehydrogenase* (*gene22236* ortholog); *FaSK*: *shikimate kinase* (*gene31604* ortholog); *FaCM*: *chorismate mutase* (*gene15010* ortholog); *FaF3D*: *flavanone 3-dioxygenase* (*gene12448* ortholog); *FaNPRL-1* (*gene20070* ortholog); *FaTGA6* (*gene14220* ortholog); *FaGrxC9* (*gene29769* ortholog); *FaWRKY51* (*gene22024* ortholog); *FaWRKY70* (*gene13547* ortholog); *FaWRKY1* (*gene07210* ortholog); *FaPR1* (*gene01774* ortholog); *FaPR5* (*gene32421* ortholog); *FaRGA1*: *resistance gene analog 1* (*gene15044* ortholog); *FaIL7R*: *interleukin-7 receptor* (*gene25131* ortholog); *FaBIRK1*: *brassinosteroid insensitive 1-associated receptor kinase* (*gene23070* ortholog); *FaPT5*: *patatin T-5* (*gene09059* ortholog); *FaH6DO*: *hyoscyamine 6-dioxygenase* (*gene01857* ortholog). The experiment was conducted in two independent trials each containing 3–4 plants per treatment per trial. Relative gene expression was measured in three technical repeats with pooled RNA from 3–4 plants. Results from two independent trials showed a similar trend, and representative data (mean ± SEM) from one trial are presented here.

To obtain a snapshot of the SA-responsive gene network in strawberry, we used the RNA pooled from 6, 12, 24, and 48 hr post-SA treatment for transcriptome analysis. About 20,000 unigenes were annotated from the transcriptomes of strawberry TY3 and SJ after SA/ddH_2_O treatment. The differentially expressed genes with predefined fold change ≥ 1.2 or ≤ 0.8 in the SA network are listed in S4–S10 Tables, and a set of 17 genes was selected from the transcriptome data and quantified by qRT-PCR (Fig 3 shows the results from one of two independent trials with a similar trend). These genes were chosen by their known functions in the SA-mediated defense pathway and their significant induction in response to SA treatment. They included four in the SA biosynthesis pathway [shikimate dehydrogenase (*FaSD*), shikimate kinase (*FaSK*), chorismate mutase (*FaCM*), and flavanone 3-dioxygenase (*FaF3D*)]; eight functioning in SA perception and signaling pathways (*FaNPRL-1*, *FaTGA6*, *FaGrxC9*, *FaWRKY51*, *FaWRKY70*, *FaWRKY1*, *FaPR1*, and *FaPR5*); the *NBS-LRR* gene *resistance gene analog 1* (*FaRGA1*); two receptor-like genes [interleukin-7 receptor (*FaIL7R*) and brassinosteroid insensitive 1-associated receptor kinase (*FaBIRK1*)]; and two substantially induced genes [patatin T-5 (*FaPT5*) and hyoscyamine 6-dioxygenase (*FaH6DO*)]. All 17 selected genes were confirmed to be SA-inducible, with most induced to relatively higher levels in the more resistant SJ than in TY3. The induction was detected as early as 6 hr after SA treatment, then decreased rapidly in both cultivars. In particular, the expression of *FaSK*, *FaCM*, *FaNPRL-1*, *FaTGA6*, and *FaRGA1* was induced at 6 and 12 hr, then returned to the basal level at 24 or 48 hr after SA treatment. Only *FaWRKY70* and *FaH6DO* remained activated in both cultivars until 48 hr after SA treatment.

### Structure features and phylogenetic analysis of strawberry NPR-like protein sequences

*NPR* genes are major components of the SA signal transduction pathway [20]. In some plants, such as Arabidopsis and grape, the expression of *NPR* genes can be activated by SA, SA analogs, and certain pathogens within a day [26, 52]. By interacting with TGA transcription factors, NPR1 and NPR3/NPR4 positively or negatively regulate the downstream defense-related genes (e.g., *PR* genes), which is essential for balancing the complex immune responses in plant cells [30, 53]. Among the five *FvNPRL* genes annotated in the diploid strawberry genome, the orthologs of *FvNPRL*-*1*, *FvNPRL-2*, *FvNPRL-3* and *FvNPRL-5* were identified in our transcriptome data, with *FvNPRL*-*1* ortholog as the only *NPRL* exhibiting significant induction after SA treatment in both TY3 and SJ (S5 Table). The *FvNPRL-4* ortholog was not detected in TY3 and SJ; this gene also showed infrequent and low expression in *F*. *vesca* [54]. The *FvNPRL-3* ortholog was annotated as a protein consisting of 591 amino acids (similar to the predicted lengths of other *FvNPRLs*) in our library but annotated as 697 amino acids in the diploid strawberry genome database. Thus, 3’ RACE was conducted to clarify the 3’-end sequences of *FvNPRL-3*. *FvNPRL-3* actually coded for a protein with 591 amino acids (S1 Fig).

**Fig 4:**
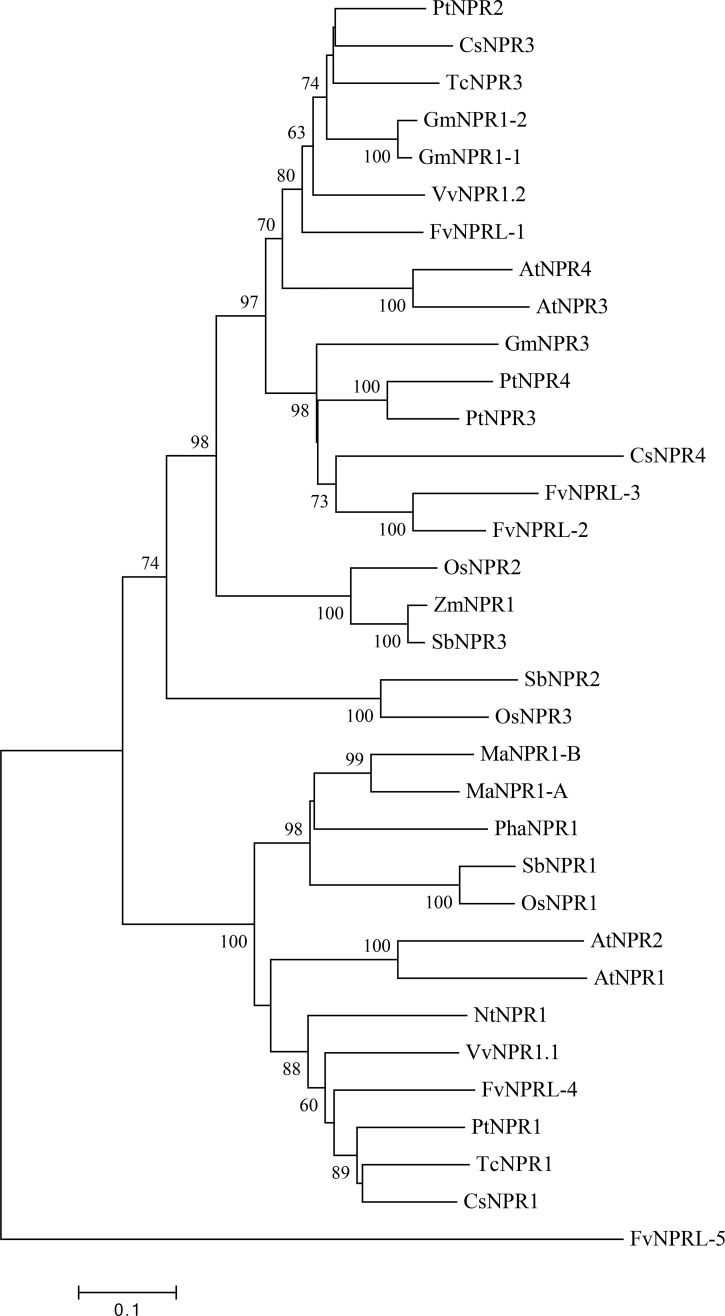
Phylogenetic analysis of NPR-like proteins. The amino acid sequences of *NPR*-like genes from *F*. *vesca* (*FvNPRL-1* to *FvNPRL-5*) were aligned with those of *Arabidopsis thaliana*, *Citrus sinensis*, *Glycine max*, *Musa acuminate*, *Oryzae sativa*, *Phalaenopsis aphrodite* subsp. *Formosana*, *Populus trichocarpa*, *Sorghum bicolor*, *Theobroma cacao*, *Vitis vinifera*, and *Zea mays*. The sequences were aligned and analyzed by the neighbor-joining method with 1000 bootstrap replications. Numbers on branches are the bootstrap values. The scale bar at the bottom indicates the evolutionary distance corresponding to 0.1 amino acid substitutions per site.

Phylogenetic analysis showed that FvNPRL-1, FvNPRL-2 and FvNPRL-3 belong to the clade containing AtNPR3, AtNPR4, TcNPR3, CsNPR3, and CsNPR4 (Fig 4). This observation suggested that FvNPRL-1, FvNPRL-2 and FvNPRL-3 may have characteristics of AtNPR3/AtNPR4. However, FvNPRL-4 was orthologous to AtNPR1, and FvNPRL-5 was not grouped into any clade. To understand the potential functions of strawberry *NPRL* genes, their amino acid sequences were aligned with AtNPR1, AtNPR3 and AtNPR4 (S1 Fig). FvNPRL-1, FvNPRL-2, FvNPRL-3, FvNPRL-4 and FvNPRL-5 all possess a conserved N-terminal BTB/POZ domain and the ankyrin repeat architecture. They also contain the conserved cysteine residues at positions 82, 150 and 160 in the AtNPR1 protein, known to be important for regulating NPR1 nuclear localization and interactions with other transcription factors in Arabidopsis [25, 55, 56]. The highly conserved NLS was found at the C-termini of FvNPRL-1, FvNPRL-2, FvNPRL-3 and FvNPRL-4, which suggests that these four NPRL proteins may translocate into the nucleus.

### Ectopic expression of *FvNPRL-1* in *A*. *thaliana* Col-0 wild type suppressed SA-mediated *PR1* gene expression and resistance to *P*. *syringae* pv. *tomato* DC3000

**Fig 5:**
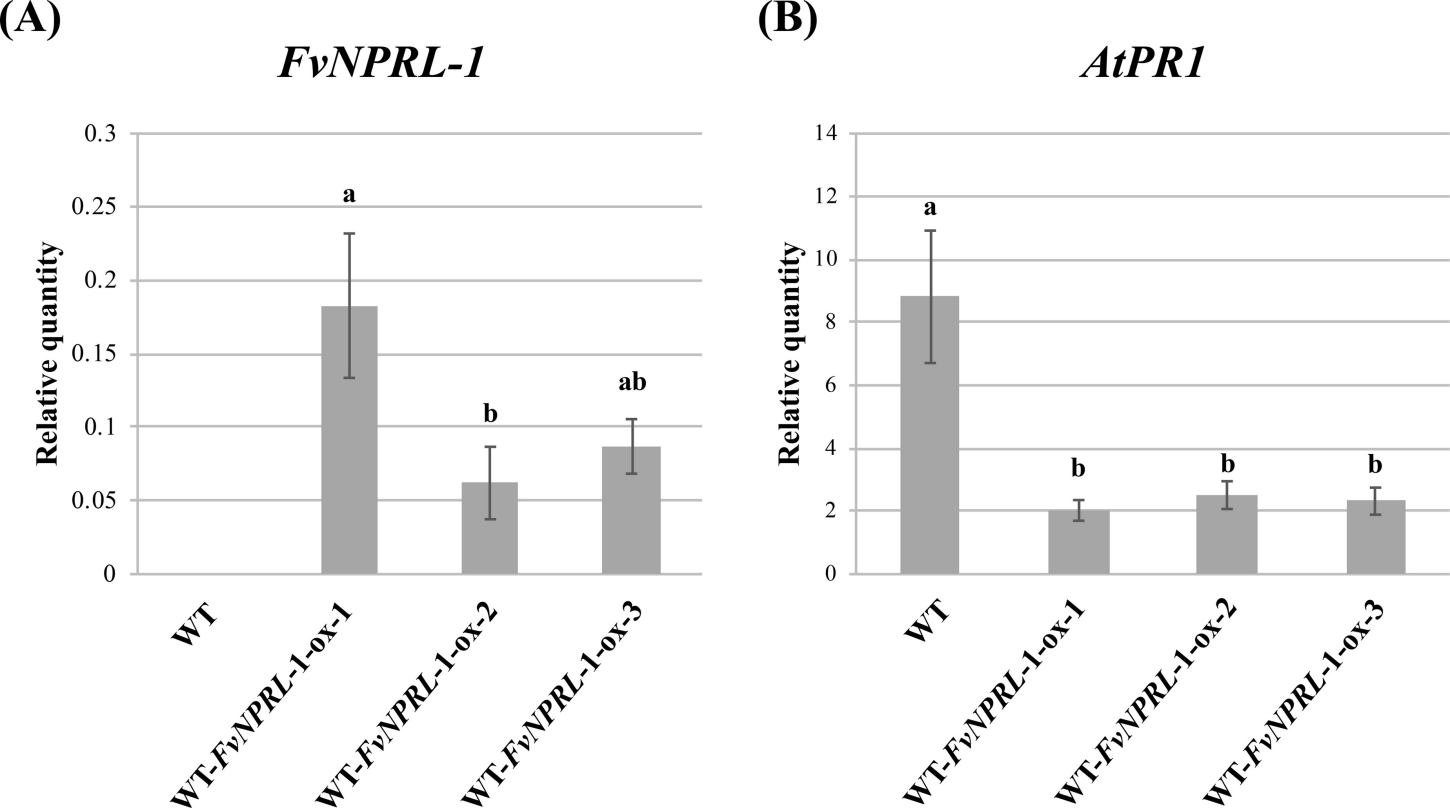
Effect of SA treatment on the expression of *FvNPRL-1* and *AtPR1* in *Arabidopsis thaliana* overexpressing *FvNPRL-1*. The expression of (A) *FvNPRL-1* and (B) *AtPR1* in response to SA treatment was evaluated in the wild type and *FvNPRL-1* transgenic Arabidopsis plants. The relative expression was the fold change in *FvNPRL-1 and AtPR1* expression compared to the internal control (*AtActin2*). Data are mean ± SEM (*n* = 3 independent trials and 3 plants per treatment per trial). Different letters indicate significant difference based on Tukey's HSD test at *p* < 0.05.

The characteristics of early induction after SA treatment (Fig 3 and S5 Table) and the conserved NPR domains suggested that *FvNPRL-1* may be a crucial regulatory component of SA-dependent defense in strawberry. To investigate whether FvNPRL-1 is functionally similar to AtNPR1, AtNPR3 or AtNPR4, transgenic lines were generated to overexpress *FvNPRL-1* driven by a 35S promoter in Arabidopsis wild type plants. *FvNPRL-1* transcripts were accumulated in leaves of homozygous T_2_ plants of wild-type/35S-*FvNPRL-1* lines 1, 2, and 3 (WT-*FvNPRL*-1-ox-1, 2, 3) (Fig 5A). To assess whether *FvNPRL-1* participates in the SA-mediated defense pathway, the downstream marker gene *AtPR1* was first quantified in the wild type and three *FvNPRL*-1 overexpression lines after 24 hr of SA treatment. The expression of *AtPR1* was significantly lower in the transgenic lines than the wild type (Fig 5B), which indicates that *AtPR1* was negatively regulated by *FvNPRL-1*.

**Fig 6:**
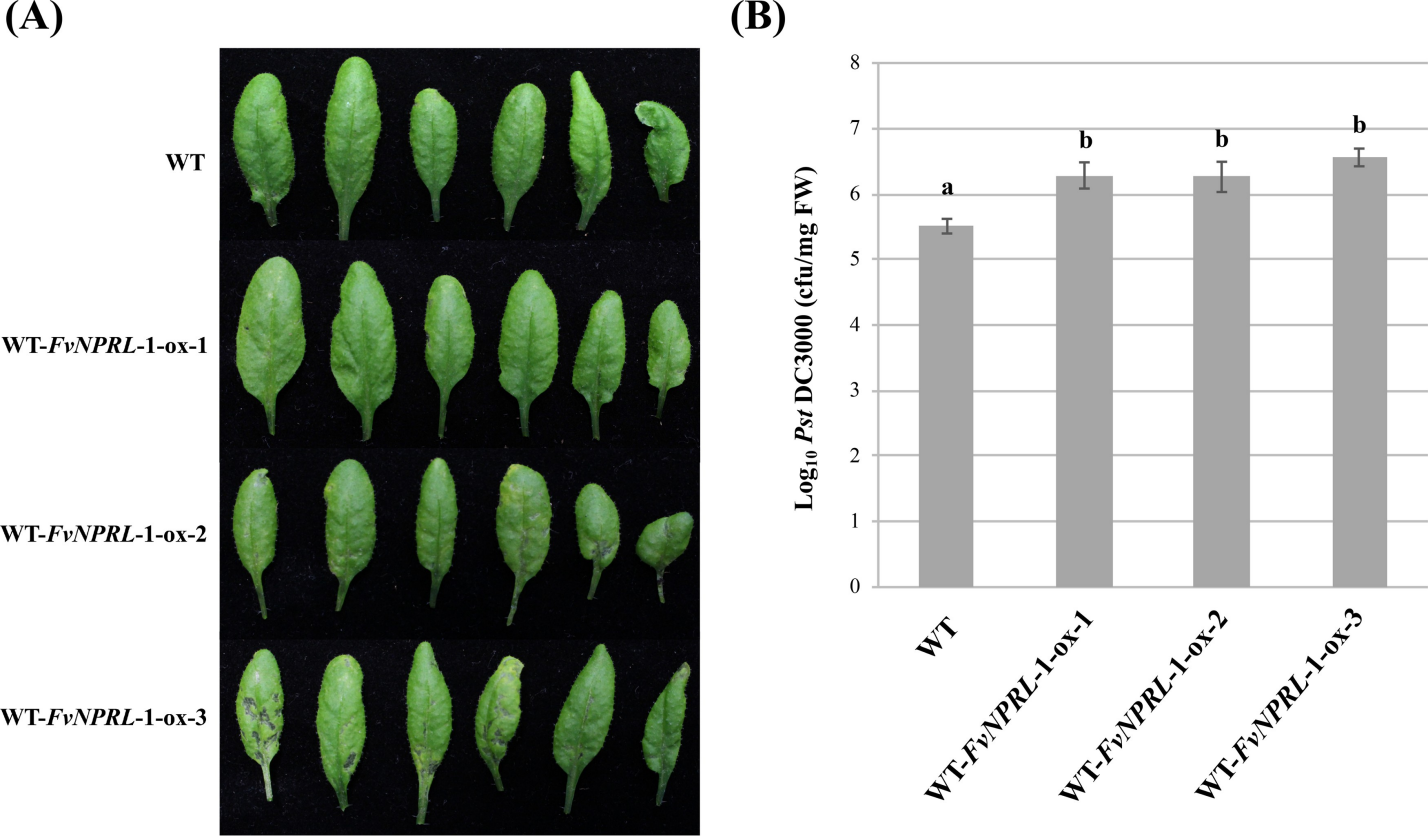
Disease development in Arabidopsis wild type and *FvNPRL-1* overexpression lines inoculated with *Pseudomonas syringae* pv. *tomato* DC3000. (A) Disease symptoms observed on day 3 after spray inoculation of 1x 10^7^ CFU/mL *Pseudomonas syringae* pv. *tomato* (*Pst*) DC3000. (B) Bacterial population of *Pst* DC3000 in leaves. WT: *Arabidopsis thaliana* Col-0 wild type; WT-*FvNPRL-*1-ox-1, 2, 3: *FvNPRL-1* overexpression lines 1, 2, 3. Data are mean ± SEM (*n* = 2 independent trials and 6 plants per treatment per trial). Different letters indicate significant difference based on Tukey's HSD test at *p* < 0.05.

The effect of *FvNPRL-1* on disease resistance was evaluated by inoculating the Arabidopsis wild type and *FvNPRL*-1 overexpression lines with *Pst* DC3000. Despite some variations, all three overexpression lines showed more severe disease symptoms and higher bacterial population (Fig 6) in leaves than did the wild type. The results indicate that *FvNPRL-1* is a suppressor of Arabidopsis resistance to the hemibiotrophic pathogen *Pst* DC3000.

### Subcellular localization of FvNPRL-1

**Fig 7:**
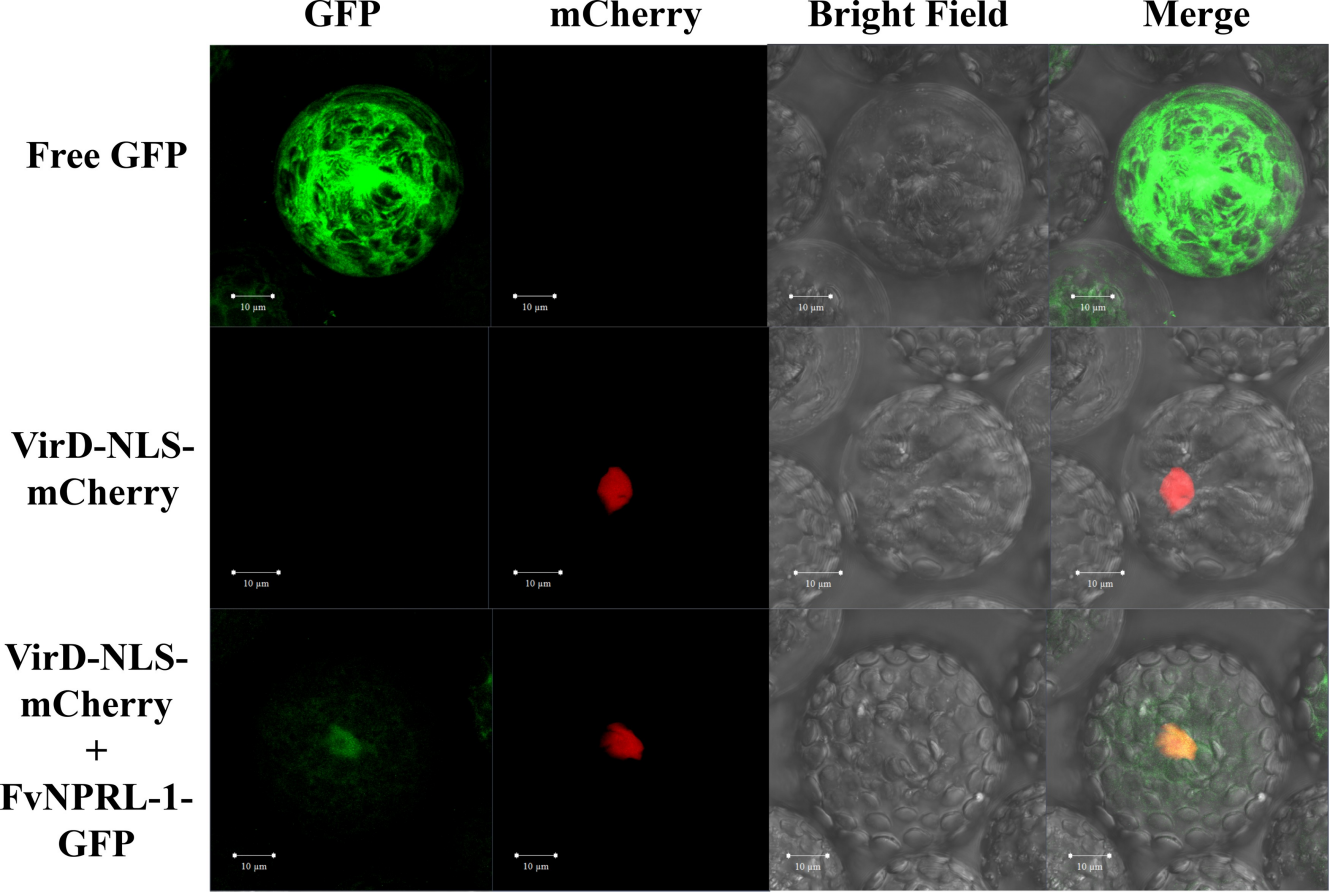
Subcellular localization of FvNPRL-1 in Arabidopsis protoplasts. Free green fluorescent protein (GFP) (upper row), VirD2-NLS-mCherry (middle row) and FvNPRL-1-GFP (bottom row) fusion protein were transiently expressed under control of the 35S promoter in protoplasts of Arabidopsis. Approximately 20% FvNPRL-1-GFP fusion protein was localized in the nucleus of protoplasts. Free GFP was a positive control, and VirD2-NLS-mCherry was a nucleus marker. Scale bars represent 10 μm.

To investigate the subcellular localization of FvNPRL-1, GFP was fused to the C-terminus of FvNPRL-1 (FvNPRL-1-GFP) for transient expression in protoplasts of Arabidopsis. Both FvNPRL-1-GFP and free GFP were observed in the cytoplasm and nucleus (Fig 7). In the normal condition (no SA), ~21% of the cells transformed with FvNPRL-1-GFP showed a GFP signal in the nucleus. The incidence of GFP signal in the nucleus was reduced to approximately 13%, 10%, and 8% after treatment with 1, 10, and 40 μM SA for 20 hr, respectively (Table 1 and S2 Fig). In contrast, the incidence of GFP signal in the nucleus remained at 58% to 63% with different SA concentrations in our positive control (free GFP) (Table 1 and S3 Fig). Thus, in Arabidopsis cells, nuclear translocation of FvNPRL-1 was negatively affected by SA.

**Table 1:** FvNPRL-1-GFP accumulated in the nucleus of Arabidopsis protoplasts under different concentrations of salicylic acid.

SA conc. (μM)	Incidence of GFP signal in the nucleus (total no. of cells)	
	FvNPRL-1-GFP	Free GFP
0	21.18% (118)	60.50% (157)
1	12.85% (140)	62.71% (118)
10	9.70% (103)	58.10% (110)
40	7.54% (106)	57.77% (97)

## Discussion

Much effort has been put into the elucidation of the SA-mediated defense network in model plants, but the hormonal modulation of immunity responses in non-model crops remains mostly unknown. In strawberry, previous defense-related studies mostly focused on diploid strawberry [57, 58] or the genes and mechanisms in *Colletotrichum*- or *Botrytis*-inoculated plants of octoploid strawberry [12, 57, 59–61]. In this study, we hypothesized that exogenously applied SA could activate strawberry defenses against anthracnose and possibly other diseases in strawberry leaves, and vital SA-mediated defense regulators could be explored in this process [11]. We directly sprayed two strawberry cultivars with SA, checked the induction of anthracnose resistance and defense marker genes, and analyzed the transcriptomes. Profiling of the SA-induced gene expression in cultivated strawberry has previously involved analysis of 1847 expressed sequence tags from whole-plant vegetative tissues at 24 hr after SA treatment [62] and by low-coverage Roche-454 sequencing (2G per cultivar) of pooled RNA samples from diverse tissues under various conditions [37]. Despite the lack of enough replicates in our transcriptome analysis, the results provide a preliminary overview of the defense network and allowed for identifying defense-related genes that are worthy of further investigation.

The SA-mediated defense mechanisms in strawberry may be similar to those in the model plant Arabidopsis. In our transcriptome data, we discovered orthologs of a variety of genes known to play critical roles in defense mechanisms as well as in SA biosynthesis, perception, and signaling in Arabidopsis and other plants [63–67]. Time-course qRT-PCR analysis of 17 selected genes validated the induced expression of 5 previously investigated marker genes (*FaGrxC9*, *FaWRKY70*, *FaWRKY1*, *FaPR1*, and *FaPR5*) [11, 12, 50, 51] in TY3 and SJ cultivars. Amil-Ruiz et al. (2016) found *FaGrxC9* (*FaGRX1*), *FaWRKY70*, *FaWRKY1*, *FaPR1*, and *FaPR5* induced at an early time point, although at a smaller magnitude, in 14-week-old plants of a strawberry cultivar Camarosa sprayed with 5 mM SA; the five genes showed increased expression at 3 days after *C*. *acutatum* inoculation. The more enhanced transient induction detected in our study may be due to different cultivars and younger plantlets (4–5 leaf stage). For the first time, the remaining 12 genes were found associated with SA-mediated defense in strawberry. These genes were induced earlier and with greater expression in the moderately resistant cultivar SJ than in TY3. This result corresponded to the study of Casado-Diaz et al. [59], in which a relatively more resistant cultivar Andana exhibited a quicker and stronger induction of defense-related genes in response to *Colletotrichum* infection.

Among the 17 differentially expressed genes, *FaSD*, *FaSK*, *FaCM*, and *FaF3D* are involved in early steps of the shikimate-phenylpropanoid pathway, from which SA and phenolic secondary metabolites are derived. Application of benzothiadiazole (BTH; chemical analog of SA) on strawberry leaves induced the accumulation of phenolics and resistance to powdery mildew [68], which supports that the shikimate-phenylpropanoid pathway is SA-responsive and defense-related in strawberry. Pattern recognition receptors (PRRs) and resistance (R) proteins recognize the elicitors or effectors of microorganisms and trigger various downstream immune responses. Treating strawberry plants with SA alone was able to activate *FaRGA1*, *FaIL7R*, and *FaBIRK1*, which implies a feedback control mechanism in the pattern-triggered immunity (PTI) and effector-triggered immunity (ETI) systems of strawberry. In Arabidopsis, positive SA regulation of certain NBS-LRR transcripts, such as RPP1 and RPS4, has been reported [69]. Among the 133 genes highly induced (fold change ≥ 5 in the transcriptome data) in response to exogenous SA in either or both of the strawberry cultivars (S10 Table), *FaH6DO* (gene01857 ortholog) and *FaPT5* (gene09059 ortholog) were confirmed by qRT-PCR. *H6DO* was found involved in alkaloid biosynthesis in a study of transgenic sugarcane lines [70], and *PT5* was reported as a potato storage protein [71]. These two genes may be new components in SA-mediated plant immunity.

*FaNPRL-1* (*FvNPRL-1* ortholog) was identified as an *NPR*-like gene significantly induced after SA treatment in the strawberry cultivars TY3 and SJ. The transcript level of *FaNPRL-1* peaked at 6 hr, remained activated at 12 hr, and returned to an inactive state at 24 and 48 hr after SA treatment. *NPR* genes were highly induced shortly after exogenous SA treatment in Arabidopsis and other plant species. For instance, the *AtNPR3*/*AtNPR4* transcripts in Arabidopsis remained at a high level from 30 min to 48 hr after treatment with SA and SA analogs such as INA and BTB [26, 72]. *TcNPR1* in cacao was dose-dependently induced at 24 hr after SA treatment [73], *GhNPR1* expression in cotton was greatly increased at 8 hr after SA treatment [74], and *MaNPR1-B* in banana was highly induced at 12 hr after SA treatment [75]. Although *FaNPRL-2*, *FaNPRL-3*, and *FaNPRL-5* seemed not significantly induced in our transcriptome data, additional experiments are required to clarify their time-course expression patterns under SA regulation.

*FvNPRL-1* was phylogenetically closer and functionally more similar to *AtNPR3*/*AtNPR4* than *AtNPR1*. In this study, the potential role of *FvNPRL-1* was characterized by ectopic expression in wild-type Arabidopsis. Such a strategy has been used to validate the similarity between *AtNPR1* and its orthologous genes in cacao [73], soybean [76] and grapevine [52]. Overexpression of *FvNPRL-1* decreased the level of *AtPR1* transcripts and increased leaf susceptibility to *Pst* DC3000. The level of increased susceptibility observed in this study was relatively minor, which may be due to different inoculation methods and the effectiveness of *FvNPRL-1* in the ectopic system. Similar disease reactions were observed when *AtNPR3* was overexpressed in wild-type Arabidopsis [77]. The overexpressed AtNPR3 and AtNPR4, coupled with TGA transcription factors, repressed the expression of WRKY70, which acts as an inducer of SA-activated genes and a repressor of JA-mediated defense [30]. As well, overexpression of *FvNPRL-1* in the Arabidopsis *npr1* mutant did not recover its resistance to *Pst* DC3000 (data not shown), so *FvNPRL-1* could not complement the function of *AtNPR1*. Taken together, similar to AtNPR3 or AtNPR4, FvNPRL-1 likely functions as a negative regulator of the SA-mediated defense. The rapid induction of *FvNPRL-1* after SA treatment implied its role as a repressor to quickly balance the effect caused by excessive SA and to fine-tune the overall defense responses.

With the C-terminal NLS, FvNPRL-1 showed the ability to translocate into the nuclei of Arabidopsis protoplasts. Similarly, AtNPR3 was found to interact with TGA2 in the nuclei of onion epidermal cells [77]. The translocation and accumulation of FvNPRL-1 seemed affected by SA concentration. Previous studies showed that SA directly interacted with AtNPR1, AtNPR3 and AtNPR4 in the nucleus and affected the resistance of Arabidopsis to pathogens [28, 31, 77]. Ding et al. also reported that SA directly suppressed the functions of AtNPR3 and AtNPR4 [30]. Although how an increase in SA suppresses the nuclear localization of FvNPRL-1 remains to be resolved, lines of evidence from previous studies suggest that plants can utilize SA to modulate the functions of NPR proteins or other regulators involved in the SA-mediated defense pathway. We need to further investigate the biological functions of FvNPRL-1 and other FvNPRLs during strawberry–pathogen interactions.

## Supporting information

S1 Fig Amino acid sequence alignment of strawberry NPR-like proteins (FvNPRL-1 to FvNPRL-5) with Arabidopsis NPR proteins (AtNPR1, AtNPR3, and AtNPR4). The conserved BTB/POZ and ankyrin repeats domains are highlighted in boxes with solid lines and dashed lines, respectively. The conserved cysteine residues are marked with black triangles. The potential nuclear localization signal (NLS) is underlined.
(TIF)

S2 Fig FvNPRL-1-GFP accumulated in the nucleus of Arabidopsis protoplasts under different concentrations of salicylic acid. FvNPRL-1-GFP fusion protein was transiently expressed in protoplasts of Arabidopsis treated with concentrations (0, 1, 10, and 40 μM) of salicylic acid. VirD2-NLS-mCherry was a nucleus marker. Scale bars represent 50 μm, and red and white arrows indicate nuclei with or without GFP signals, respectively.
(TIF)

S3 Fig Free GFP accumulated in the nucleus of Arabidopsis protoplasts under different concentrations of salicylic acid. Free green fluorescent protein (GFP) fusion protein was transiently expressed in protoplasts of Arabidopsis treated with concentrations (0, 1, 10, and 40 μM) of salicylic acid. VirD2-NLS-mCherry was a nucleus marker. Scale bars represent 50 μm, and red and white arrows indicate nuclei with or without GFP signals, respectively.
(TIF)

S1 Table Scoring scale for strawberry anthracnose. (DOCX)

S2 Table Primers used for qRT-PCR on strawberry. (DOCX)

S3 Table Primers used for transgenic Arabidopsis and 3' RACE. (DOCX)

S4 Table Genes involved in SA biosynthesis pathway (RNAseq analysis). (XLSX)

S5 Table Genes encoding NPR, TRX/GRX, TGA and WRKY proteins (RNAseq analysis). (XLSX)

S6 Table Genes encoding pathogenesis-related (PR) proteins (RNAseq analysis). (XLSX)

S7 Table Genes encoding resistance (R) proteins (RNAseq analysis). (XLSX)

S8 Table Genes encoding pattern recognition receptor (PRR) proteins (RNAseq analysis). (XLSX)

S9 Table Genes involved in CDPK/MAPK cascade (RNAseq analysis). (XLSX)

S10 Table Highly induced genes (fold change ≥ 5 in either or both of Taoyuan 3 and Superjumbo cultivars). (XLSX)
