# Supplementary figures and images for: Identification of a strawberry NPR-like gene involved in negative regulation of the salicylic acid-mediated defense pathway
Source: PLoS One. 2018 Oct 12;13(10):e0205790. doi: 10.1371/journal.pone.0205790 (PMC6185849; doi:10.1371/journal.pone.0205790)

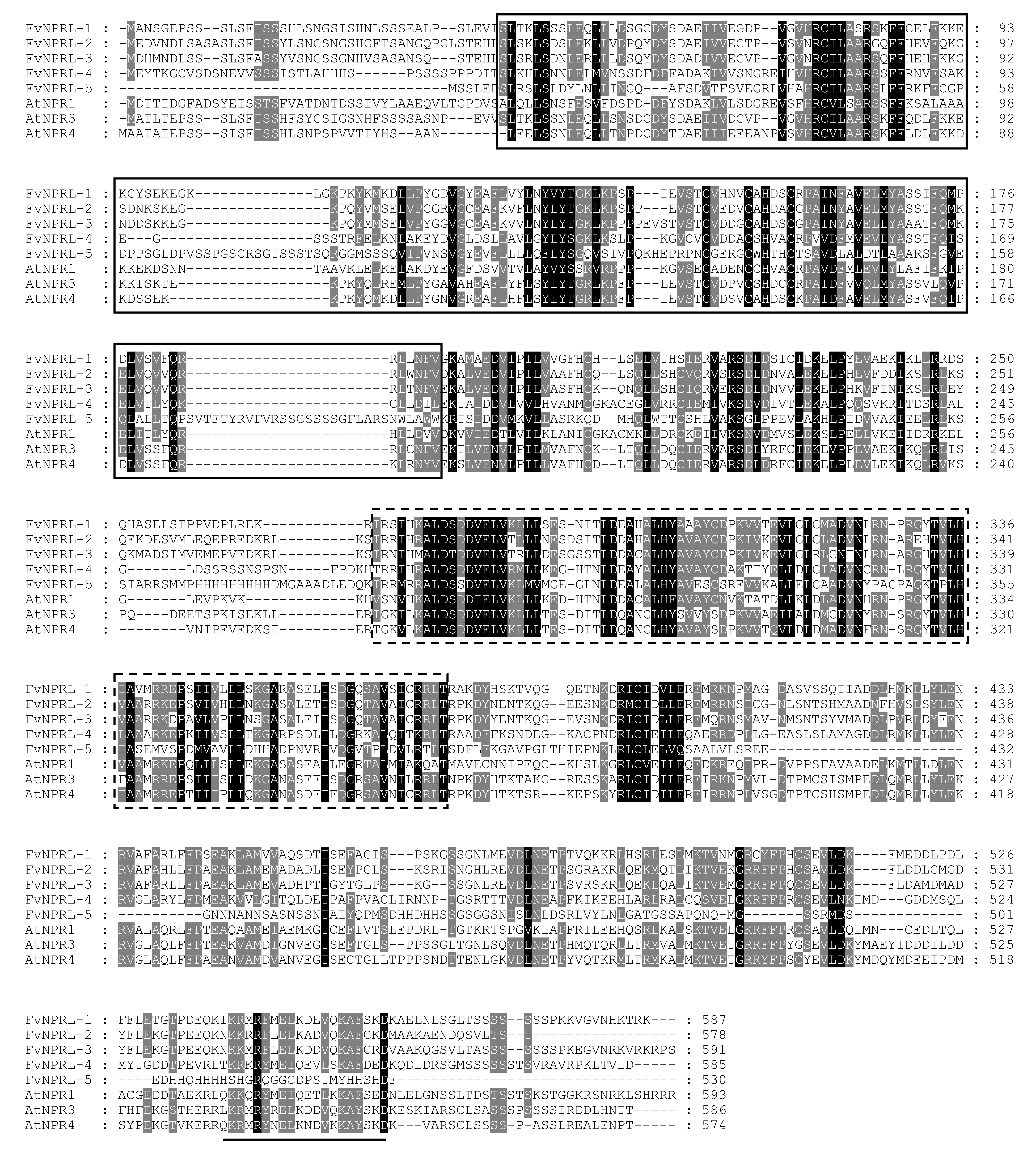

Supplement: S1 Fig — The conserved BTB/POZ and ankyrin repeats domains are highlighted in boxes with solid lines and dashed lines, respectively. The conserved cysteine residues are marked with black triangles. The potential nuclear localization signal (NLS) is underlined. (TIF) [file pone.0205790.s001.tif]

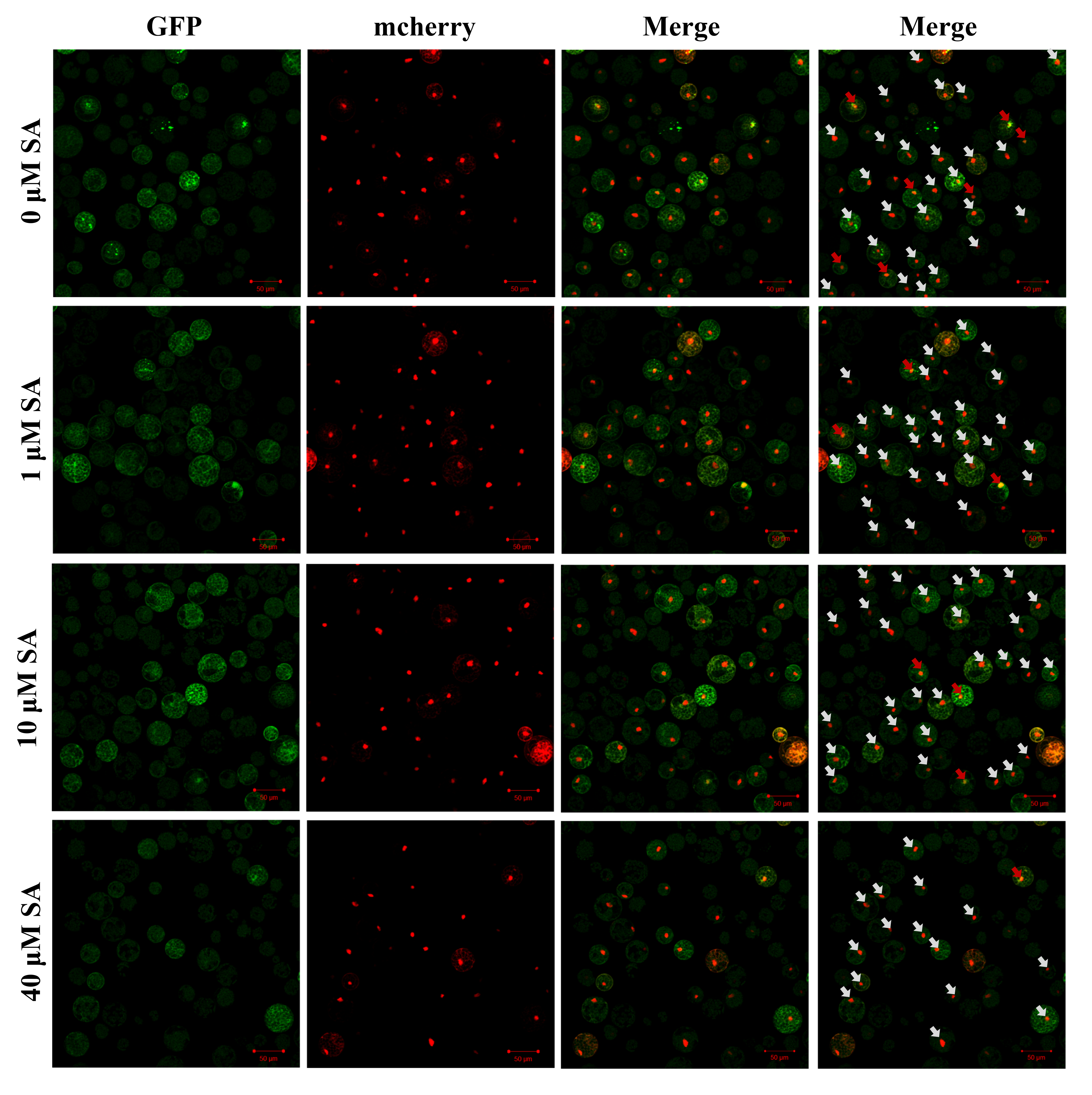

Supplement: S2 Fig — FvNPRL-1-GFP fusion protein was transiently expressed in protoplasts of Arabidopsis treated with concentrations (0, 1, 10, and 40 μM) of salicylic acid. VirD2-NLS-mCherry was a nucleus marker. Scale bars represent 50 μm, and red and white arrows indicate nuclei with or without GFP signals, respectively. (TIF) [file pone.0205790.s002.tif]

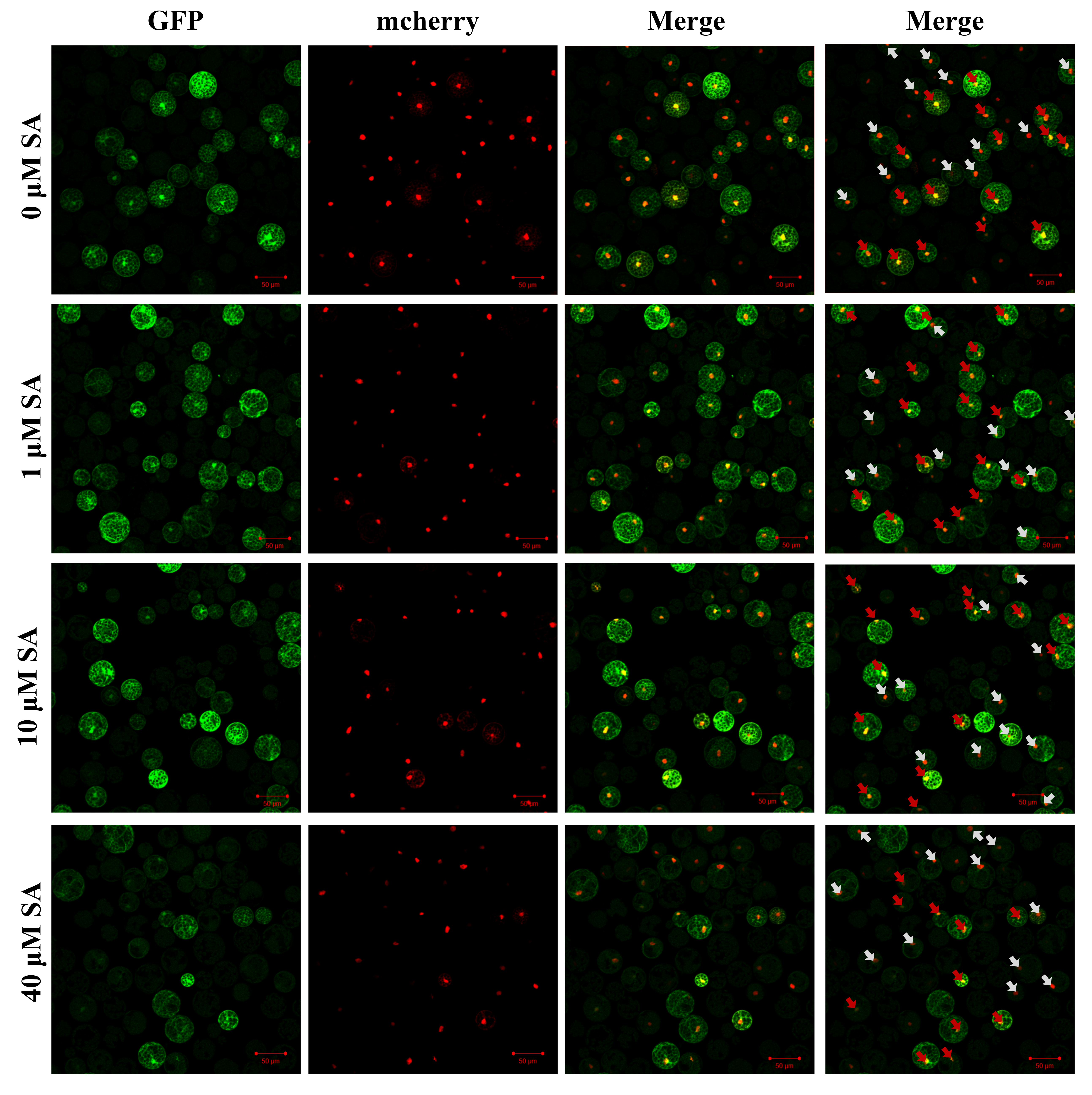

Supplement: S3 Fig — Free green fluorescent protein (GFP) fusion protein was transiently expressed in protoplasts of Arabidopsis treated with concentrations (0, 1, 10, and 40 μM) of salicylic acid. VirD2-NLS-mCherry was a nucleus marker. Scale bars represent 50 μm, and red and white arrows indicate nuclei with or without GFP signals, respectively. (TIF) [file pone.0205790.s003.tif]
