# Supplementary material for: Identification of a strawberry NPR-like gene involved in negative regulation of the salicylic acid-mediated defense pathway
Source: PLoS One. 2018 Oct 12;13(10):e0205790. doi: 10.1371/journal.pone.0205790 (PMC6185849; doi:10.1371/journal.pone.0205790)
Supplement: S1 Table — (DOCX) [file pone.0205790.s004.docx]

**S1 Table. Scoring scale for strawberry anthracnose**

| **Scale** | **Description of Symptoms** |
| --- | --- |
| **1** | Lesions only on juvenile leaves; lesion size < 0.5 mm; lesion number < 5 |
| **2** | Lesions only on juvenile leaves; lesion size ~1 mm; lesion number < 5 |
| **3** | Lesions only on juvenile leaves; lesion size ~1 mm; lesion number 5-10 |
| **4** | Lesions on juvenile and mature leaves; lesion size ~1 mm; lesion number > 10 |
| **5** | Lesions on juvenile and mature leaves; lesion size between 1-2 mm; lesion number > 10; red and black spots on the petiole |
| **6** | Lesions on juvenile and mature leaves; lesion size between 1-2 mm; lesion number > 10; black spots on the petiole |
| **7** | Lesions on juvenile and mature leaves; lesion size > 2 mm; evident black spots on the petiole; some lesions slightly depressed |
| **8** | Lesions on juvenile and mature leaves; lesion size > 2 mm; depressed black spots on the petiole |
| **9** | Coalesced lesions on the leaves; leaves dehydrated and slightly wilted; lesions on the petiole cause the leaf to drop |
| **10** | Wilted and dead plantlet |
