# Supplementary material for: Identification of a strawberry NPR-like gene involved in negative regulation of the salicylic acid-mediated defense pathway
Source: PLoS One. 2018 Oct 12;13(10):e0205790. doi: 10.1371/journal.pone.0205790 (PMC6185849; doi:10.1371/journal.pone.0205790)
Supplement: S2 Table — (DOCX) [file pone.0205790.s005.docx]

**S2 Table. Primers used for qRT-PCR on strawberry**

|  | 1. **Gene Annotation** | | | | | |  | | 1. **Amplicon** | | |  | |  | |
| --- | --- | --- | --- | --- | --- | --- | --- | --- | --- | --- | --- | --- | --- | --- | --- |
| 1. **Gene Name** | 1. **Accession no. from NCBI database** | | 1. **Gene ID in the diploid genome** | | 1. **Identity percentage to the diploid genome** | |  | | 1. **Position** | | 1. **Fragment Size (bp)** | 1. **Primer Sequence** | | 1. **References** | |
| 1. ***FaActin*** | 1. ref\|XM_004304313\| | | 1. gene22626 | | 1. 70% | |  | | 1. 318~428 | | 1. 111 | 1. F:CCTCACAGAAGCACCCCTTA 2. R:GAAAGGACAGCCTGAATTGC | | 1. Zhang and Shih (2007) | |
| 1. ***FaOLP2* (*FaPR5*)** | 1. ref\|XM_004298732.1\| | | 1. gene32421 | | 1. 99% | |  | | 1. 278~401 | | 1. 124 | 1. F: CGGCGGCAGACAGCTTAA 2. R: TGCCCGCATTCACGTCTAG | | 1. Zhang and Shih (2007) | |
| 1. ***FaWRKY1*** | 1. ref\|XM_004310052.1\| | | 1. gene07210 | | 1. 99% | |  | | 1. 333~440 | | 1. 108 | 1. F: TGACGATGGATACAGGTGGA 2. R: TGCTTCTTCACATTGCAACC | | 1. Encinas-Villarejo et al. (2009) | |
| 1. ***FaPR1*** | 1. ref\|XM_004289651.1\| | | 1. gene01774 | | 1. 99% | |  | | 1. 183~380 | | 1. 125 | 1. F: TGCTAATTCACATTATGGCG 2. R:TTAGAGTTGTAATTATAGTAGG | | 1. Grellet-Bournonville et al. (2012) | |
| 1. ***FaSD*** | 1. ref\|XM_004302432.1\| | 1. gene22236 | | 1. 99% | |  | | 1. 1662~1769 | | 1. 107 | | 1. F: AGACTCCTCCGTGAAGCAAA 2. R: GCTGGCAAACCAGTGAAGTT | 1. This study | |  |
| 1. ***FaSK*** | 1. ref\|XM_004302183.1\| | 1. gene31604 | | 1. 99% | |  | | 1. 692~792 | | 1. 100 | | 1. F: CCTCATGCTCCACGAATGTA 2. R: GTTGGTATGATGGGGTCTGG | 1. This study | |  |
| 1. ***FaCM*** | 1. ref\|XM_004291909.1\| | 1. gene15010 | | 1. 99% | |  | | 1. 1071~1191 | | 1. 120 | | 1. F: GGCAAGAAGTGAGCCTTGAC 2. R: GAGGCATAACCCATTCTCCA | 1. This study | |  |
| 1. ***FaF3D*** | 1. ref\|XM_004307866.1\| | 1. gene12448 | | 1. 80% | |  | | 1. 718~828 | | 1. 130 | | 1. F: AACACATTGGGAGAGCAAGG 2. R: CGAGGTCTTGGAGGAGAATG | 1. This study | |  |
| 1. ***FaNPR1*** | 1. ref\|XM_004293686.1\| | | 1. gene20070 | | 1. 98% | |  | | 1. 1513~1651 | | 1. 139 | 1. F: TGAACTGAGCACTCCACCTG 2. R: AGCATGGGCTTCATCTAAGG | | 1. This study | |
| 1. ***FaGrxC9*** | 1. ref\|XM_004293851.1\| | | 1. gene29769 | | 1. 99% | |  | | 1. 297~429 | | 1. 132 | 1. F: CGCTGTTGTCGTTGAGCTAC 2. R: GTCGCCATAACCTTCTCCAA | | 1. This study | |
| 1. ***FaWRKY51*** | 1. ref\|XM_004288505.1\| | 1. gene2202 | | 1. 96% | |  | | 1. 314~445 | | 1. 131 | | 1. F: TGATTGTGCATTCCCTCGTA 2. R: GTGAAGAACAGCCCAAATCC | 1. This study | |  |
| 1. ***FaWRKY70*** | 1. ref\|XM_004302544.1\| | 1. gene13547 | | 1. 96% | |  | | 1. 562~676 | | 1. 114 | | 1. F: ATGAATCAGGGCCAGCAGTA 2. R:TGAGTGGGAGTCAAGAGCCTA | 1. This study | |  |
| 1. ***FaTGA6*** | 1. ref\|XM_004307039.1\| | 1. gene14220 | | 1. 98% | |  | | 1. 539~635 | | 1. 96 | | 1. F: TCCCCACTAACTGCTGCTCT 2. R: GGTGTTTCTTGTGGCTTGGT | 1. This study | |  |
| 1. ***FaRGA1*** | 1. ref\|XM_004291888.1\| | 1. gene15044 | | 1. 99% | |  | | 1. 1502~1614 | | 1. 113 | | 1. F: ATGTCTTGCGAGACCTTGCT 2. R: ATGCCTGTCCCATTCTCTTG | 1. This study | |  |
| 1. ***FaBIRK1*** | 1. ref\|XR_184595.1\| | 1. gene23070 | | 1. 95% | |  | | 1. 401~506 | | 1. 106 | | 1. F: TGTGGGATTCAGTGAAGGTG 2. R: GAGCCATCATTCACCAGACA | 1. This study | |  |
| 1. ***FaIL7R*** | 1. - | 1. gene25131 | | 1. 98% | |  | | 1. 96~211 | | 1. 116 | | 1. F: GATTGGAGGAGTGGTTATGG 2. R: TGAGAGAGCATCGCTTCAGA | 1. This study | |  |
| 1. ***FaH6DO*** | 1. ref\|XM_004309465.1\| | 1. gene01857 | | 1. 76% | |  | | 1. 52~195 | | 1. 144 | | 1. F: GTCCCTTCAAACTACATTCG 2. R: TTGGGCAATCTGTTGGATAA | 1. This study | |  |
| 1. ***FaPatatin T-5*** | 1. ref\|XM_004291083.1\| | 1. gene09059 | | 1. 99% | |  | | 1. 37~133 | | 1. 97 | | 1. F: CCCAGTCATGGAAAGCTCAT 2. R: GCTCGGACTCTAGGAAGCTG | 1. This study | |  |
