# Supplementary material for: Identification of a strawberry NPR-like gene involved in negative regulation of the salicylic acid-mediated defense pathway
Source: PLoS One. 2018 Oct 12;13(10):e0205790. doi: 10.1371/journal.pone.0205790 (PMC6185849; doi:10.1371/journal.pone.0205790)
Supplement: S3 Table — (DOCX) [file pone.0205790.s006.docx]

**S3 Table. Primers used for transgenic Arabidopsis and 3' RACE**

| **Name** | **Primer sequence** | **Note** | References^a^ |
| --- | --- | --- | --- |
| ***AtActin2*** | F: AGGTCCAGGAATCGTTCACAGA  R: CCCCAGCTTTTTAAGCCTTTGA | qRT-PCR on Arabidopsis | Chen et al. (2014) |
| ***AtPR1*** | F: GTCTCCGCCGTGAACATGT  R: CGTGTTCGCAGCGTAGTTGT | qRT-PCR on Arabidopsis | Chen et al. (2014) |
| ***FvNPRL-1*** | F: GGGCCATGGATGGCGAATTCAGGTGAGC  R: GGGCACGTGCTATTTTCTAGTCTTGTGATTTAC | Underline: *NcoI* site  Underline: *PmacI* site | This study |
| ***FvNPRL-1*** | F: GGGGACAAGTTTGTACAAAAAAGCAGGCTTCACCATGGCGAATTCAGGTGAGC  R: GGGGACCACTTTGTACAAGAAAGCTGGGTGTTTTCTAGTCTTGTGATTTAC | Underline: attB1  Underline: attB2 | This study |
| **3'RACE adapter** | R: GCGAGCACAGAATTAATACGACTCACTATGGTTTTTTTTTTTTVN | 3' RACE | Shaefer (1995) |
| **3'RACE outer** | R: GCGAGCACAGAATTAATACGA CT | 3' RACE | Shaefer (1995) |
| **3'RACE inner** | R: CGCGGATCCGAATTAATACGACTCACTATAGG | 3' RACE | Shaefer (1995) |
| ***FvNPRL-3* 2nd exon** | F: TGATGCTTGTGCTCTCCACT | 3' RACE | This study |
| ***FvNPRL-3* 4th exon** | F: CTTCTTCCCCCAATGTTCA | 3' RACE | This study |

^a^References:

Chen CC, Chien WF, Lin NC, and Yeh KC. Alternative functions of *Arabidopsis Yellow Stripe-Like3*: from metal translocation to pathogen defense. PLoS One 2014;9(5)**:**e98008. doi: 10.1371/journal.pone.0098008. PubMed PMID: 24845074.

Schaefer BC. Revolutions in rapid amplification of cDNA ends: new strategies for polymerase chain reaction cloning of full-length cDNA ends. Anal Biochem 1995;227(2)**:**255-273. doi: 10.1006/abio.1995.1279. PubMed PMID: 7573945.
